# Medical News and Miscellany

**Published:** 1890-06

**Authors:** 


### Medical News and Miscellany

DR. H. K. LEAKE, of Dallas, left for Europe on the 12th. He goes to Birmingham to study gynecology, and will be with Mr. Tait all summer.

PROF. F. L. SIM, of the Memphis Medical Monthly was elected President of the Association of American Medical Editors at the recent Nashville meeting. Good!

PROF. EMORY LAMPHEAR has been elected to the chair of Orthopædic Surgery in the Kansas City Medical College. This flourishing School had 31 matriculants for fall course, as early as June 1.

DR. E. J. BEALL, of Fort Worth, left on 10th inst., for Europe, via New York. He will attend the meeting of the British Medical Association at Birmingham, and later (August) the International Medical Congress at Berlin.

DR. W. B. ANDERSON has removed from Rices' Crossing, Williamson county, to Llano; Dr. H. S. Robertson, from Lockhart to Yoakum; Dr. J. M. Borders, from Sexton to Fort Worth; Dr. Z. T. Bundy, from Midlothian to Waxahachie.

---

THE advertisement of the Chattanooga Medical College which appeared first in our May number, was received too late to be "noticed." We call attention to it now, and advise students to investigate the claims of this Southern school before matriculating. Chattanooga is certainly a delightful place in summer and winter; and to study there, would be a luxury.

---

THE REPORTER TAKLES THE MEDICAL PROFESSION, AND MAKES "HASH."—It is not to be supposed that the average reporter is quite up to all the jaw-breaking words used by the average doctor; but with the courteous aid of an able-bodied (?) Secretary, to help him spell, and to give him points, it would seem that the reporter would make a little closer *guess* at what is going on, whereof he essays to "report," than the following: (this is copied *verbatim* from the Fort Worth——paper.)

"Dr. E. J. Ward, of Waxahachie, read a peculiar and able paper, entitled "Is Dentition Invention to be used in the Treatment of Placenta Prævia?"

[The title was—"Is dentition a factor in the production of infantile diseases?"]

We learn that the reporter soaked his head two hours that night, and thus refreshed, felt equal to next day's battles. So he reports:

"Dr. R. H. L. Bibb who exhibited a trahlectomy tube wherein it had remained for a long time in the Section on Surgery was on motion requested to prepare his explanation of the same for reference to the publishing committee."

[Dr. Bibb exhibited a tracheotomy tube, taken from the trachea of a patient, where, through negligence of a former attendent, it had been left, and the "exhibition" took place in the Section on Surgery.]

*This reporter, we learn, is still at large.*

THE PATIENCE OF JOB—couldn't stand it. Doctor Merryman was telling Bennett and other Austin physicians who did not attend the meeting, all about it, and amongst other things said: "*Danels* was as polite as a dancing master. With one eye on the speaker during a discussion, and one hand taking down his remarks—he managed to answer fifty thousand questions at the same time, all on different subjects,—and he gave every one a patient, thorough courteous and satifactory answer every time. I never saw him lose his patience but once."

"When was that," said Bennett.

"It was after adjournment, when he was trying to straighten up the papers on his desk, preparatory to going to dinner. While beset with reporters, and by members asking "if their applications had been acted on," and "what had become of their diplomas," and numerous other questions that, really, he *ought* to answer; at the same time dictating for two or three reporters—and as hungry as —well, as hungry as he *looks*," said the fat old rogue, shaking his round sides. "He had given the reporter everything asked for, and *spelled* it for him; till finely—

'What was name of the doctor that was elected First Vice-President and resigned?' said the sad-eyed reporter.

"That was Dr. Fort, of Paris; Dr. J.—M.—Fort," said the Secretary, patiently, but with a suppressed yawn.

"Doctor—H. M. Post, did you say, Doctor?"

"No! Doctor—J—M—Fort—!"

"How do you spell Fort?" said the indefatigable pencil shover.

"Then *Danels* let down. He fell into a chair, and fanning himself with the last paper read in the last Section—replied—'F! O! R! T!—FORT! ! My *lord*, man! *how else* WOULD or COULD you spell Fort?'

"I have he-aird it spelt with a P!" said the reporter.

---

AN INDICATORY ROSTRTM.—Dr. F. J. Lutz, (St. Louis Courier of Medicine) speaking of the recent meeting of the Missouri Medical Association says: "I believe the rostrum will show that about 175 members registered."

We would like to "get onto" that arrangement in operation in the Missouri Medical Association, by which the rostrum is made to indicate how many members registered. At our meetings the difficulty is to get members to register; out of the estimated five hundred in attendance at Fort Worth, first and last, seventy-eight by actual count, registered their names. Now, if the "rostrum" could be made not only to make members register, but to show afterwards, how many registered, a rostrum should become a part and parcel of every Secretary's outfit. We presume the rostrum of the Missouri Medical Association arranged to show ever so long after the meeting the number of members who were present and had registered, was furnished by the arrangement committee; (or is it a patent?) if so, Brother Sears, at Waco, Chairman of the Arrangement Committee for our next meeting, should enter into correspondence with the Secretary of the Missouri Medical Association with a view of borrowing it, or of getting one like it. We are a little puzzed to understand this thing. Perhaps all the members (175) of the Missouri Medical Association were assembled at one time on the rostrum and an instantaneous photo was taken of them? No, this would have shown how many were present on the rostrum at one time; but how did it show how many had registered? Don't you suppose Brother Lutz meant "roster?"

